# Understanding Anterior Cruciate Ligament Adaptation: Structural, Mechanical, and Healing Considerations

**DOI:** 10.1007/s40279-026-02498-1

**Published:** 2026-08-07

**Authors:** Oskar K. Staniucha, Samantha M. Birse, Daniel E. Ferris, Bradley A. Morris, Ayden M. McCarthy, Tim J. Gabbett, Robert G. Crowther

**Affiliations:** 1 School of Science and Technology, University of New England, Armidale, NSW 2350 Australia; 2 Sports Performance, Exercise and Activity Research (SPEAR), University of New England, Armidale, NSW 2350 Australia; 3 Gabbett Performance Solutions, Brisbane, QLD 4011 Australia

## Abstract

The anterior cruciate ligament (ACL) is both a passive stabiliser and an active mechanoresponsive tissue. Emerging evidence demonstrates that its morphology, material properties, and microstructure adapt to the mechanical milieu imposed by habitual activity, targeted exercise, and surrounding musculature. This narrative review synthesises animal and human evidence on ACL mechanobiology, highlighting how loading parameters, maturational timing, and neuromuscular context shape ligament structure, function, and injury risk. At the molecular level, the ACL responds to mechanical loading through differential collagen isoform expression, matrix remodelling, and angiogenic signalling. These processes are further modulated by sex and maturation, with males typically demonstrating greater upregulation of type I collagen, whereas females synthesise comparatively less. Regional vascularity also influences adaptive capacity, potentially constraining remodelling within the mid-substance, where injury most frequently occurs. Animal ligament research shows that endurance and resistance training can increase stiffness, tensile strength, and cross-sectional area, while human studies report ligament hypertrophy in athletes who begin high-load training during adolescence. Conversely, chronic underloading leaves the ACL undersized, whereas poorly directed or chaotic overload fosters micro-damage accumulation and fatigue failure. Integrating resistance training with variable and sport-specific movement practice enhances ligament resilience and reduces initial and reinjury risk. Collectively, the ACL should be regarded as a mechanoresponsive tissue the capacity of which is determined by the interplay of morphology, vascularity, mechanotransduction, sex, maturation, and load history. An integrated load–adaptation–injury framework is proposed to guide future research and optimise clinical prevention and rehabilitation strategies.

## Key Points

**Table:** 

The anterior cruciate ligament is a dynamic, load-responsive tissue that adapts to mechanical stress through changes in structure and strength.
Appropriate and progressive loading, particularly during adolescence, may enhance ligament resilience, while both underloading and excessive or poorly controlled loading may increase injury risk.
Aligning strength and neuromuscular training with ligament biology may help optimise adaptation and support long term knee health.

## Introduction

Ligaments are dense bands of connective tissue that connect bones across joints, providing mechanical stability, guiding motion, and contributing to proprioception [1]. Ligaments are primarily composed of collagen type I and III fibres aligned in parallel bundles, interspersed with fibroblasts, elastin, glycoproteins and proteoglycans [2–4]. This organisation enables the ligament to resist tensile forces along the direction of load while allowing limited elasticity to accommodate small joint excursions [1, 5]. Their low vascularity and cellular turnover result in a slow adaptive response to mechanical loading, which is sensitive to load magnitude, frequency, and recovery [6–8]. Repetitive submaximal loading from daily physical activities, resistance training, or sport can induce ligament micro-trauma [9, 10]. Micro-trauma can be defined as microscopic tearing of tissue, potentially promoting adaptation (or maladaptation) over time [11]. In contrast, macro-trauma results from a single high-magnitude loading event that exceeds tissue capacity, resulting in acute injury [12]. The anterior cruciate ligament (ACL) exemplifies this risk, being susceptible to micro- and macro-trauma in athletic or occupational contexts [9, 13].

ACL ruptures are among the most devastating injuries in sport, accounting for approximately 6% of all injuries and a reinjury rate of 17–23% in elite sporting environments [14–16]. Complete rupture is associated with prolonged recovery periods (6–12 mths) and substantial economic costs, estimated at $30,000 AUD per case [14, 17, 18]. Additionally, ACL injuries frequently result in chronic conditions such as osteoarthritis, occurring in approximately 30% of cases [19].

While ligaments have traditionally been viewed as biologically inert, a growing body of mechanobiology research suggests otherwise. The ACL is capable of hypertrophy, extracellular matrix (ECM) remodelling, and alterations in tensile properties in response to controlled mechanical loading [13, 20–23]. However, it remains vulnerable to fatigue failure under high-frequency or unpredictable multidirectional loading, where cumulative micro-damage exceeds its repair capacity [24, 25]. Despite this interplay between adaptation and vulnerability, ligament responses are comparatively underrepresented in broader load-management frameworks, which tend to focus instead on muscle, tendon, cartilage, and bone [26]. As a result, there is a need to better understand the dose–response relationship between exercise load, muscular capacity, and ligament adaptation to inform both injury prevention and rehabilitation strategies.

In this review, we examine the structural and mechanical characteristics of ACLs and synthesise current evidence on their adaptive responses to loading. We discuss how load type, magnitude, frequency, and recovery influence ligament health, plasticity, and injury risk. PubMed and Scopus were searched using combinations of the following terms: “anterior cruciate ligament”, “ACL”, “strain”, “exercise”, “adaptation”, “injury”, “rehabilitation”, and “recovery”. Searches were conducted in October 2025, with backward and forward citation tracking performed on included studies to ensure comprehensive coverage. We included English-language, peer-reviewed studies involving human participants and animals, and manually screened systematic reviews to identify additional primary sources. Evidence was interpreted with consideration of study design, methodological rigour, sample characteristics, and consistency of findings, with greater weight given to studies of higher methodological quality and relevance where findings conflicted. Our aim is to assist clinicians, researchers, and performance practitioners in integrating ligament-specific principles into holistic training and rehabilitation strategies.

## Imaging, Structure, and Composition of the ACL

Magnetic resonance imaging (MRI) is typically used for in-vivo assessment of ligamentous structures with 3 Tesla (T), semi-automated algorithms, and diffusion tensor imaging (DTI) enabling detailed quantification of ACL volume, cross-sectional area (CSA), and fibre anisotropy [27–29]. MRI T2/T1ρ relaxation mapping, volumetry, and MRI shear-wave elastography now allow researchers to estimate ex vivo ligament stiffness and demonstrate moderate predictive relationships in cadaveric specimens (R^2^ ≈ 0.60) with mechanical failure thresholds when combined with ligament volume, imaging parameters and ligament type [30]. While these approaches remain largely limited to experimental settings, advances in MRI-based techniques may offer the potential for longitudinal, in-vivo monitoring of ligament adaptation and graft maturation. However, translation into routine practice will depend on ecological validity, cost, accessibility, and whether these methods provide clinically actionable information beyond existing assessment approaches.

Although these advances have improved the capacity to characterise ACL morphology and infer aspects of tissue behaviour, their translational value requires careful consideration. Most advanced imaging approaches remain costly, technically demanding, and concentrated within specialised research or tertiary imaging centres, which may limit accessibility and broader clinical uptake. In addition, the ecological validity of static or highly controlled imaging assessments must be considered when interpreting a structure that functions under complex, dynamic, and sport-specific loading conditions. Accordingly, the clinical impact of these approaches will depend not only on their sensitivity to structural change, but also on whether they offer accessible, cost-effective, and decision-relevant information that improves risk stratification, monitoring, or rehabilitation beyond existing clinical assessment methods.

Using these techniques, studies have identified morphological factors associated with ACL injury risk. Notably CSA, length, volume, and insertional geometry are consistently reported as morphological features of tensile capacity and ligament stability [31–33]. The ACL reaches peak CSA at approximately 37–39% of its length from the tibial insertion, a region corresponding to the greatest mechanical demand due to combined tensile and shear loading during cutting and landing tasks [34]. CSA is influenced by body mass in males and intercondylar notch width in females [35]. Normative CSA values range from 33–83 mm^2^ in adult males and 24–58 mm^2^ in adult females, often diverging by mid-adolescence [35].

Beyond gross size and insertional geometry, the ACL comprises two functional bundles, the anteromedial (AM) and posterolateral (PL), which differ in orientation and tensioning behaviour across knee flexion [36, 37]. The AM bundle remains taut throughout flexion and is primarily responsible for resisting anterior tibial translation, whereas the PL bundle is maximally tensioned in near-extension and contributes strongly to rotational stability [37–39]. Injury patterns often involve disproportionate strain on the PL bundle during pivoting or cutting manoeuvres performed at shallow knee angles, while the AM bundle experiences higher tensile load when anterior tibial shear force is present during deep knee flexion tasks [36, 40]. Reconstruction techniques that fail to restore both bundles have been linked to persistent rotational instability, highlighting their complementary roles in joint mechanics [41]. Understanding bundle-specific function is therefore critical, not only for surgical restoration, but also for exercise prescription. Given the AM bundle’s role in resisting anterior tibial translation at greater knee flexion angles, strengthening strategies targeting the hamstrings and soleus may contribute to posterior shear forces that help modulate loading in this range. In contrast, as the PL bundle is more highly strained near extension and under rotational loads, neuromuscular strategies that emphasise landing and cutting mechanics, including increased knee and hip flexion, controlled deceleration, and reduced dynamic valgus and tibial internal rotation, may help limit strain in these positions. However, the extent to which targeted interventions may selectively influence bundle-specific loading during dynamic tasks remains uncertain and findings should be interpreted with caution.

While these MRI-derived morphological features are increasingly used to identify structural risk factors, most assessments occur after injury or reconstruction. As such, little is known about ACL morphology in its pre-injury state, and findings from retrospective comparisons may not reflect true predispositions. Moreover, inconsistency in imaging protocols, segmentation techniques, and measurement landmarks may potentially limit the reproducibility of reported values [35, 42]. This methodological variability hinders cross-study comparisons and weakens the foundation for using morphology as a clinical or screening tool. Despite these methodological limitations, a clear understanding of the ACL’s structural makeup and underlying biological mechanisms remains essential for interpreting morphological variation and adaptation from guided exercise prescription.

Structurally, the ACL comprises predominantly type I collagen fibres (~ 90% of dry weight), interwoven with elastin, type III collagen, proteoglycans (e.g., decorin, biglycan), and glycoproteins such as periostin and thrombospondins [2, 22, 43]. Together, these components confer anisotropic mechanical properties, providing high tensile strength along the fibre axis while enabling viscoelastic responses under cyclic loading [44]. Cross-link density and collagen crimp patterns further modulate ligament stiffness, strain-rate sensitivity, and susceptibility to plastic deformation [5, 45]. Notably, the ACL's limited vascularity and heterogeneous perfusion constrain its capacity for nutrient diffusion and tissue turnover, especially in the mid-substance where vascular supply is lowest [8, 13, 46].

Together, the ACL’s macroscopic geometry and ECM composition form a structure to resist anterior tibial translation and multiaxial shear forces. However, these structural features have the capacity for adaptation. Evidence from elite athletic populations and animal models indicates that ligament morphology is load-sensitive, particularly during adolescence [20, 21, 47, 48]. When examining morphological changes, exploratory evidence suggests sex-based differences in collagen mRNA expression following ACL injury, with males demonstrating greater type I collagen expression [49]. Additionally, recent work in animal models indicates that female ACL tissue may accumulate greater collagen matrix damage under equivalent loading conditions and exhibit a slower reparative response compared to males [50].

In the absence of direct compositional data in the ACL, insight can be drawn from tendon models, where a predominance of type III collagen, characterised by a more disorganised and multidirectional fibre orientation, may compromise tissue organisation and mechanical strength [51]. While direct evidence in the ACL is limited, similar compositional shifts may influence ligament integrity, increasing susceptibility to injury and potentially affecting rehabilitation and return-to-sport outcomes. This response may also represent an adaptive strategy to accommodate greater multidirectional shear loading, potentially offering a different mode of joint protection. Given this capacity for mechanical and biochemical adaptation, the ACL is increasingly recognised as a mechanoresponsive tissue.

## Mechanobiology of ACL Adaptation

Mechanical stimuli initiate transcriptional and translational responses such as upregulation of collagen type I and III, matrix metalloproteinases, and angiogenic factors, which collectively modulate ECM composition, vascular plasticity, and overall tissue integrity [52–56]. These processes are further shaped by regional vascularity, hormonal influences, and developmental timing, meaning that the adaptive capacity of the ACL is not uniform across individuals or life stages [9, 13, 57]. Understanding these cellular and molecular pathways is essential for explaining variability in ligament remodelling and provides the foundation for interpreting how mechanical load translates into structural adaptation. These responses are likely mediated by mechanical strain, linking tissue-level deformation and subsequent morphological changes. While localised strain is a key driver of tendon adaptation [58, 59], its role in the ACL remains less clearly defined and may vary according to regional structure and loading distribution.

Short-term high-intensity treadmill running in rats has been shown to upregulate over 120 ACL proteins, including collagens IX, XIV, and XVIII, lumican, periostin, and transforming growth factor (TGF)-β, without inducing histological damage [10]. This profile reflects increased biosynthetic and ECM remodelling activity and is specific to the ACL, as no such response was observed in the adjacent medial collateral ligament (MCL), displaying a tissue-specific mechanoresponse. The ACL’s ability to remodel is not determined by biosynthetic activity alone but is also shaped by its structural and vascular architecture.

Adaptation is constrained by vascular supply [6, 9]. The ACL receives blood from the middle and inferior geniculate arteries, yet perfusion is regionally heterogeneous. The distal and proximal regions exhibit higher vascular density, while the mid-substance is relatively avascular [8, 46]. This pattern limits nutrient diffusion and regenerative capacity in the central region, where injury most often occurs [60]. In contrast, the MCL displays more uniform vascularity and a more robust angiogenic response post-injury [6, 7, 61], contributing to its greater healing profile. These differences suggest that vascular architecture directly influences a ligament’s capacity to remodel in response to load.

Animal models provide strong evidence that mechanical loading enhances the structural and mechanical properties of the ACL. Endurance running increases ACL stiffness and failure load by 15–25% in 6 wk old rats [47]. Similarly, resistance-wheel training in adolescent mice enhances ACL tensile strength, alters tibial slope geometry, and increases ACL width and CSA [48, 62]. These findings suggest that loading during growth may influence ligament integrity as well as the development of surrounding bony structures. Although animal models cannot directly replicate human ligament behaviour, they may provide the groundwork for exploratory studies to be done on human participants, particularly given reported similarities in ligament mechanical properties across species (i.e., swine, goat, cow, rat) [63].

Human cross-sectional studies currently provide preliminary evidence that ACL morphology may be associated with exposure to mechanical loading. Weightlifters who began training during pre- or peri-puberty demonstrated greater ACL CSA compared to untrained controls [21]. Additionally, greater ACL size has been observed in the dominant limbs of figure skaters and springboard divers, independent of training years [20]. These findings parallel tendon adaptations to long-term loading [64–66], suggesting that ligaments may exhibit load-related structural differences in a load-specific and limb-dependent manner.

Adaptive responses within the ACL may also be modulated by sex. Exploratory evidence from injured ACL tissue suggests females demonstrate lower type I gene expression compared to males, suggesting divergent pathways for balancing stiffness with compliance under load [49]. As type I collagen is the primary tensile load-bearing component of the ACL, this difference may have implications for tissue repair capacity and mechanical resilience, although its significance for intact ligament adaptation remains unclear. Importantly, ACL adaptation is governed by a complex interplay between vascular architecture, sex-specific molecular pathways, and the magnitude and nature of mechanical loading. However, the relative composition of collagen subtypes within the ACL remains poorly understood, particularly in relation to sex-based differences.

While ligament mechanotransduction initiates the cellular basis for adaptation, these molecular events occur within a broader mechanical context. The downstream load-response process unfolds across multiple timescales, beginning with rapid gene expression and extending to long-term structural reorganisation [67–71]. In vivo and ex vivo rabbit models show that the ACL can alter gene expression within hours of cyclic loading, reflecting immediate mechanosensitive signalling rather than structural change [68, 70]. These short-term responses may reflect early signalling processes associated with matrix remodelling, although their translation to human ligament may be limited. In partial-injury models, upregulation of matrix metallopeptidase-13 (MMP-13; collagenase-3), a member of the matrix metalloproteinase family involved in extracellular matrix degradation, and α-smooth muscle actin (α-SMA), a marker of the contractile potential of ligament cells such as myofibroblasts, occurs at 1 wk [67]. This early response precedes increased expression of collagen type I alpha-1 (COL1A1) and collagen type III alpha-1 (COL3A1) by 6 wks, marking the transition from degradation to synthesis [67].

Despite these insights, the current understanding of ligament recovery is largely inferred from other soft-tissue models. Ligaments are often assumed to follow a 24–72 h recovery window, similar to tendon or muscle, yet direct evidence for this timeframe is limited. Much of the long-term knowledge on structural remodelling and vascular maturation derives from graft studies, where “ligamentisation” describes tendon-to-ligament transformation under load [69, 72]. Extrapolating these findings to native tissue should therefore be approached with caution.

Collectively, these findings indicate that ligament adaptation represents a continuum, beginning with rapid gene activation and culminating in delayed structural reorganisation, occurring in response to the magnitude, direction, and frequency of mechanical loading. This continuum forms the physiological foundation through which external muscle forces and strength capacity ultimately dictate ACL loading dynamics.

## Muscle Forces, Strength, and ACL Loading

The mechanical environment of the ACL is shaped primarily by the surrounding musculature [73]. While the intrinsic structural properties of the ligament set its tolerance to strain, it is the neuromuscular system that dictates the magnitude, direction, and timing of external forces applied across the joint [73–75]. Recognising this interaction is essential for interpreting injury mechanisms, identifying modifiable strength imbalances, and designing targeted preventative interventions that address both tissue and movement demands.

Biomechanical models show that quadriceps and gastrocnemius activation elevate anterior tibial shear forces, particularly when the knee is flexed less than 50°, potentially increasing ACL strain during landing, cutting, and deceleration tasks [73, 75, 76]. Quadriceps activation may either increase or reduce ACL loading depending on joint position and co-contraction, with more extended, quadriceps-dominant postures linked to higher-risk movement patterns [73, 76–78]. However, ACL strain is task- and individual-dependent, influenced by movement mechanics (kinematics), strength characteristics (e.g., hamstring-quadriceps ratio), and muscle activation timing [79, 80], and does not directly translate to injury risk. Instead, ACL injury risk is more strongly associated with movement patterns that exhibit dynamic knee valgus and poor neuromuscular control, rather than isolated muscle activation [81].

Weight-bearing tasks such as single-leg landing, drop vertical jump, and sidestep cutting expose the knee to combined anterior shear, joint compression, and multiplanar knee moments, including valgus and tibial internal rotation loading [77, 81, 82]. ACL injury risk typically arises when these loads occur simultaneously, particularly in extended knee postures with limited neuromuscular control, as demonstrated in cadaveric and modelling studies [73, 76, 83–85]. During cutting and deceleration tasks, insufficient hamstring activation relative to quadriceps demand may limit the capacity to restrain anterior tibial translation. Poor gluteal control can increase dynamic knee valgus and transverse plane loading, further elevating ACL strain [81, 86, 87]. In contrast, coordinated activation of the hamstrings, soleus, and hip abductors during these tasks contributes to posterior shear, joint compression, and frontal plane control, collectively reducing ACL loading [73, 88].

The hamstrings apply posteriorly directed forces that counteract anterior translation, with this protective effect most pronounced beyond 20–30° of knee flexion [89, 90]. They may also contribute to reducing ACL loading through their influence on transverse (i.e., tibial internal rotation) and frontal plane knee mechanics (i.e., knee valgus), in addition to opposing anterior shear forces [73]. The soleus, although it does not cross the knee joint and is unaffected by knee flexion angle, also contributes substantial posterior shear forces at the tibia. It may further influence tibiofemoral loading through its contribution to joint compression and ground reaction forces during dynamic tasks [73, 76]. Its stabilising role is influenced instead by ankle position, where dorsiflexion generates passive tension and plantarflexion produces high active forces, both of which help unload the ACL [73]. The gluteus medius and minimus also contribute by reducing valgus loading, a common injury mechanism during lateral movement and cutting [73, 86].

Strengthening of the posterior chain, including the hamstrings, gluteals, and plantar flexors, improves joint stability by lowering peak knee abduction and internal rotation moments during dynamic tasks such as landing, cutting, and deceleration [91–93]. Higher quadriceps torque, particularly at 60°·s⁻^1^, has been positively associated with greater ACL CSA [94], suggesting a potential link between muscular strength and ligamentous hypertrophy. However, direct prospective evidence linking isolated strength measures to reduced ACL injury incidence remains limited, with most support arising from associations with biomechanical risk factors. Despite this, disproportionately high quadriceps strength relative to the hamstrings can elevate anterior shear forces and strain the ACL, particularly during knee extension phases of movement [73, 75, 95].

ACL strain peaks at knee flexion angles less than 30°, making the ligament particularly vulnerable during landing and cutting [73, 75, 95]. At these acute angles, quadriceps contraction generates anterior tibial shear forces even with submaximal torque output, supported by in vivo and biomechanical investigations across healthy and rehabilitation populations, and experimental models of ACL loading[83, 96]. These mechanics replicate high-risk postures, highlighting the need to assess joint-specific strength. For example, assessing at 30° of knee flexion combined with 45° of hip flexion isolates posterior chain contributions, including hamstring length–tension capacity and soleus-mediated restraint [90, 97, 98]. Yet, many existing isometric testing protocols use seated or prone postures that maximise peak torque but lack ecological validity, leaving a gap within the literature regarding ACL injury mechanics.

Targeted training at specific joint angles identified as high risk for ACL rupture is an emerging strategy to reduce injury risk during common movement tasks in sport (e.g., jumping, landing, and cutting) that are known to increase ACL strain [20, 99]. Non-contact ACL injuries typically occur at acute knee flexion angles (~ 11° to 30°), often during the initial contact phase of jumping, landing, or cutting [100]. Exposing the ligament to controlled submaximal loads within this range, through a combination of isometric, low-velocity, and high-velocity contractions, may stimulate mechanotransducive signalling without exceeding fibre strain thresholds. Importantly, direct human evidence remains limited, and thresholds distinguishing adaptive from injurious loading are not yet well defined. In parallel, teaching stiff-landing mechanics with high plantar-flexor engagement, while avoiding flat-foot or heel-first contacts, may redirect force absorption to the ankle complex and reduce proximal ligament loading [101, 102].

Together, these insights emphasise the importance of muscular coordination, joint-angle specificity, and balanced strength development in ACL protection and potential adaptation. However, the extent to which such interventions influence ligament adaptation remains unclear. While strengthening the posterior chain is often advocated to reduce ACL loading, it may also alter joint torque distribution with unclear implications for both loading and adaptation. Current evidence does not establish the type, magnitude, frequency, or recovery window of loading required to optimise ACL adaptation, particularly across key maturational stages. This knowledge gap highlights the need for research that considers growth-related changes, biological sex, and the developmental mismatch between muscle and ligament capacity when evaluating both injury risk and adaptive potential.

## Developmental and Sex-Specific Factors

Ligament loading is governed by both muscular coordination and structural tolerance, and its capacity shifts across maturation. Pubertal growth alters the relative timing of musculoskeletal development and neuromuscular control, creating windows where strength and coordination lag behind rapid changes in body size and tissue properties [103, 104]. This mismatch places disproportionate forces on immature joints and soft tissues, magnifying the mechanical stress at the knee, particularly the ACL [86, 105, 106]. Peak height velocity (PHV), the period of maximal growth rate in stature, is commonly used as a non-invasive measure of biological maturation and to approximate this window of ACL rupture vulnerability [107]. The inextricable link between pubertal development, musculoskeletal growth, and neuromuscular control introduces critical windows of vulnerability, particularly for adolescent females, who exhibit a greater number of ACL injury risk factors such as higher knee valgus angles, reduced hamstring activation, and quadriceps-dominant strategies during landing and cutting tasks, than adolescent males [108, 109]. These sex-specific and age-dependent differences in ACL structure and function may help explain variations in load tolerance and injury risk.

Increased transverse-plane moments and greater body mass elevate ACL forces during landing tasks, with these effects being markedly higher in late- and post-pubertal females compared to their pre-pubertal comparisons [110]. The ACL’s ability to resist these rising loads is strongly influenced by its stiffness, viscoelasticity, CSA and structural size [34, 111]. Yet, ACL growth largely plateaus around age 10 yrs, creating a discrepancy between rising load demands and relatively fixed tissue capacity [112]. This loading-capacity mismatch is compounded by oestrogen-mediated laxity and an attenuated neuromuscular spurt, referring to the comparatively limited gains in strength, coordination, and joint stabilisation during puberty [104, 113]. Epidemiological data indicate that ACL injury incidence is similar between boys and girls during pre-puberty, with rates estimated at 0.5–1.5 injuries per 10,000 athletic exposures [88, 114]. However, following PHV, incidence diverges sharply [105, 115]. This divergence is preceded by a rapid acceleration in injury rates during late childhood and early adolescence, with the steepest increase observed between 5 and 14 yrs (~ 10.4% per year) [89]. Adolescent females aged 15–19 yrs exhibit a 3–sixfold higher risk than their male counterparts, with reported rates up to 5.7 per 10,000 athletic exposures in high-school sports [89, 108, 109]. This sex-specific increase coincides with developmental mismatches between muscle strength, ligament growth, and neuromuscular control, compounded by hormonal influences on tissue laxity and motor coordination [104, 105, 113]. In contrast, males typically experience more favourable scaling of muscle mass and ligament CSA during adolescence, which appears protective [116–118]. These differences should not be viewed as purely biological, as neuromuscular and biomechanical disparities between females and males are reduced when skill level, strength, and training age are matched [119]. Differences in exposure, conditioning history, and access to training during development may also contribute to ACL loading and injury risk [120]. These epidemiological patterns highlight the importance of maturational timing in shaping injury risk and suggest early implementation of targeted neuromuscular interventions may be warranted.

Hormonal influences may further modulate developmental and sex-specific vulnerabilities. Oestrogen can reduce collagen cross-linking and increase ligament laxity, particularly during menstrual cycle peaks [113], while cortisol may impair collagen synthesis under stress or inadequate recovery [121]. In contrast, insulin-like growth factor 1 (IGF-1) promotes collagen synthesis and muscle hypertrophy, promoting ligament protection [53]. Similarly, testosterone plays a key role in fibroblast activity, muscular hypertrophy, and strength [122–124]; however, testosterone displays no stimulation of collagen synthesis [125]. Despite these systemic effects, few studies have directly examined how endocrine profiles shape ACL morphology in vivo. This gap is especially relevant in adolescent females, in whom fluctuating oestrogen and progesterone may interact with smaller ligament size and delayed neuromuscular development to elevate risk, while males benefit from more stable testosterone-driven connective tissue growth [122–124].

Sex-based differences in ACL morphology further influence risk. Males tend to exhibit larger CSA, particularly within the distal 41% of the ligament, whereas in females, CSA is more closely associated with intercondylar notch width [34]. A narrower notch reduces the space for ligament excursion and may increase impingement risk during dynamic valgus. These morphological disparities persist even when normalised to body size, suggesting underlying developmental determinants rather than simple anthropometric scaling.

Importantly, muscular development does not always track with ligament growth. Longitudinal evidence shows that between ages 9 and 15 yrs, girls’ quadriceps strength increases more rapidly than hamstrings strength, while ACL dimensions exhibit little growth [126]. This disproportion may amplify ACL strain, particularly during landing and cutting tasks, which impose multiplanar demands rather than isolated shear forces [73, 84, 85]. Targeted hamstring and soleus conditioning during puberty may help offset the imbalance between quadriceps and hamstring strength, and ACL growth by enhancing posterior directed force-generating capacity [73]; however, the extent to which these adaptations translate to altered movement strategies and reduced ACL loading in dynamic tasks remains unclear and findings should be interpreted with caution. Emerging evidence suggests that strength development may contribute to motor learning and movement refinement, although these relationships are complex and not fully understood [127]. Moreover, quadriceps strength itself is not inherently detrimental, and its relationship with ACL loading is highly context-dependent, influenced by joint kinematics and neuromuscular coordination. In addition, repeated exposure to submaximal ligament strain through improved muscular control may stimulate mechanotransducive pathways that support beneficial structural adaptation within the ligament [20, 21, 47, 58]. Collectively, these developmental and sex-specific factors highlight the importance of timing in both injury risk and preventative training.

## Movement Mechanics and Injury Risk

ACL injury risk during dynamic tasks is strongly influenced by movement strategy, particularly when joint loading exceeds the ligament’s mechanical tolerance [77, 81, 128]. Established biomechanical risk factors include dynamic knee valgus, reduced knee flexion angles, and poor trunk control, which collectively contribute to high-risk loading patterns [77, 81, 128]. Key kinematic factors such as lower-limb and trunk angles, together with kinetic variables including ground-reaction force vectors and joint moments, interact to determine joint loading profiles associated with ACL injury risk. Higher stance-phase knee-abduction moments have been prospectively linked to future ACL injury in adolescent female athletes, although these findings are considered highly task- and population-dependent and should not be interpreted as universal injury thresholds [81]. Myer et al. [87] derived a 25.25 Nm cut-point for knee abduction moment during the drop-vertical jump from the female cohort data of Hewett et al. [81], using it to classify athletes as high- or low-risk for ACL injury. However, subsequent work has highlighted variability in lower-limb kinematics across commonly used screening tasks and populations, limiting the generalisability of such thresholds [77], and emphasising the limitations of dichotomising a continuous relationship between load and injury risk [129]. Normative movement patterns vary substantially across tasks and individuals, with systematic reviews demonstrating wide variability in kinematics, further limiting the utility of fixed cut-points for injury prediction [77]. Furthermore, video-based analyses of ACL injury mechanisms indicate that injury typically occurs under combined multiplanar loading conditions during sport-specific manoeuvres, rather than isolated threshold exceedance [130, 131]. Exceeding this load likely pushes the ACL into the non-linear region of its force–length curve, where fibre strain becomes uneven and failure risk rises [74, 132]. As such, laboratory-derived biomechanical markers are better interpreted as indicators of loading tendencies rather than definitive predictors of injury risk.

Landing and surface characteristics also contribute to ACL loading profiles. Movement patterns involving reduced hip and knee flexion, flat-foot landings, or excessive foot pronation increase anterior shear and valgus torque at the knee. Surface-specific comparisons suggest higher ground-reaction forces on artificial turf than on natural turf [133, 134]. Footwear choice is another external factor, with low-support athletic footwear shown to modestly increase ACL loading in late-pubertal females [135]. These factors may compound underlying neuromuscular deficits during high-velocity tasks.

Trunk mechanics further influence knee loading. Poor trunk stability alters centre-of-mass trajectories and increases frontal and transverse-plane moments at the knee, elevating valgus and internal rotation stresses [82, 136]. Interventions that enhance trunk stability and reactive control have been shown to reduce these loads, potentially lowering ligament strain during athletic movements [137, 138]. Poor trunk control often manifests as dynamic valgus, a combined knee abduction and internal rotation pattern closely linked to non-contact ACL injury mechanisms [139]. Collectively, these biomechanical factors highlight the pathways by which injury risk emerges and ways in which it may be reduced. Structured resistance training, neuromuscular instruction, and gait retraining represent practical means of addressing these deficits, forming the foundation of exercise interventions designed to improve ACL health.

## Exercise Interventions to Optimise ACL Health

Targeted exercise remains the most direct and controllable factor influencing ACL loading, mechanical resilience, and recovery outcomes. The structural integrity of the ligament is constrained by developmental and biological factors. However, appropriate exercise and training interventions can promote positive adaptations in both tissue and movement mechanics.

Resistance training (RT) is a central modality yet remains under prescribed in clinical rehabilitation for post-ACL injury [140]. This under-prescription applies not only to the frequency of RT but also to the intensity prescribed. A 2021 systematic review found that RT protocols post-ACL reconstruction rarely exceeded 60% of one-repetition maximum (1RM), despite evidence that such intensities fall short of restoring the force demands of sport [140, 141]. Trials employing higher loads, including ≤ 6RM schemes, have not demonstrated adverse effects on graft laxity [140], supporting their safety and potential necessity for ligament and tendon adaptation.

Beyond strength, motor learning strategies influence how load is applied during sport-specific movements. Training approaches characterised by high variability, such as non-linear pedagogy or differential learning, expose athletes to constantly changing movement demands [142, 143]. This variability promotes adaptability by encouraging exploration of multiple motor solutions, which improves robustness under unpredictable sport conditions [142]. As a result, athletes trained in this way demonstrate lower valgus and rotational loads during cutting tasks compared with those receiving traditional linear instruction [143]. Instructional focus also shapes outcomes, with external cues (e.g., ‘touch the cone softly’) consistently outperforming internal cues (e.g., ‘keep your knee over your toe’) in promoting movement patterns that reduce ACL strain [144].

Building on these principles, structured neuromuscular training (NMT) programs, such as FIFA 11 + , Prevent Injury and Enhance Performance (PEP), and Knee Control, have consistently demonstrated 30–50% reductions in ACL injury incidence across youth and adolescent cohorts, particularly in female athletes [145–149]. These programs typically integrate lower-limb strength training, plyometrics, dynamic balance, and movement-technique instruction, with emphasis on knee-over-toe alignment and soft-landing mechanics [149]. However, as many are delivered as warm-up programs, they also do not meet minimum strength training thresholds, particularly with respect to load intensity. Further, structured NMT programs may not substantially alter external biomechanics [150]. Despite strong evidence for efficacy, compliance and long-term adoption remain variable, and mechanobiological underpinnings are rarely addressed. By framing NMT within a load-adaptation model, clinicians may refine these interventions to not only reduce acute injury risk but also foster long-term ligament adaptation. For example, incorporating high-intensity posterior-chain isometrics or controlled loading at vulnerable flexion angles may more directly stimulate mechanotransducive pathways while preserving the motor learning benefits of variability and external cueing.

Movement variability may also protect ligament and joint health. Reduced knee kinematic variability following ACL reconstruction has been linked to poorer femoral cartilage proteoglycan density [151], suggesting that restoring natural fluctuations in gait and landing patterns may prevent focal overload. Gait retraining programmes that reintroduce controlled variability could therefore serve a dual function, promoting joint longevity while reducing recurrent ligament loading.

Collectively, these approaches offer a multidimensional framework to enhance ACL capacity, from structural adaptation to improved neuromuscular control. By addressing both tissue resilience and movement quality, such interventions may reduce injury risk and support long-term joint health. The following section integrates these concepts into a unified framework linking load, adaptation, and injury risk across the full spectrum of activity.

## An Integrated Load-Adaptation-Injury Framework

**Fig. 1:**
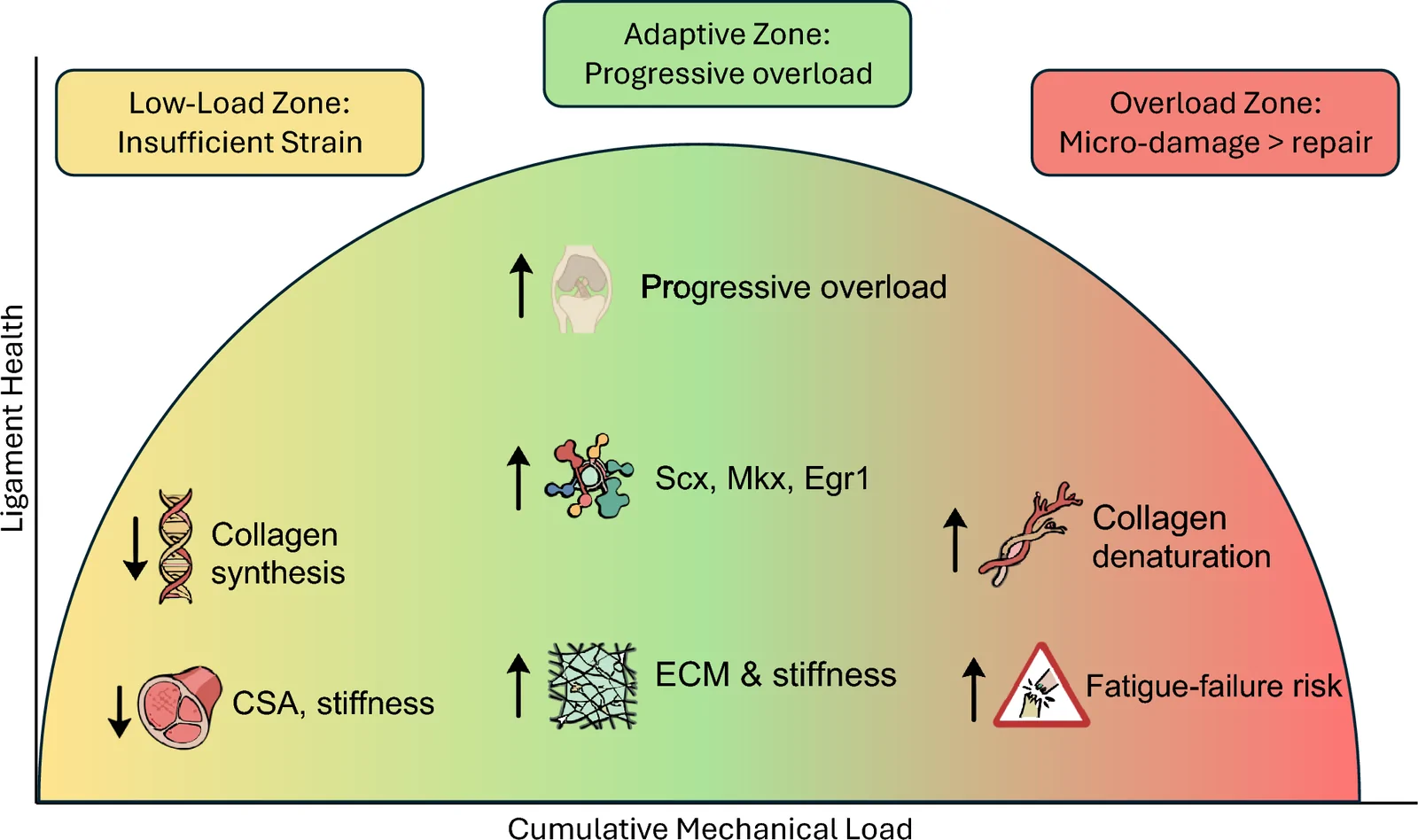
Proposed Inverted U-shaped load-adaptation-injury framework. *CSA* cross-sectional area; *Scx* scleraxis; *Mkx* mohawk homeobox; *Egr1* early growth response 1; *ECM* extracellular matrix

Building on established dose–response concepts in biology, rehabilitation, and sports science, we propose a provisional ACL-specific load-adaptation framework based on an inverted U-shaped relationship between cumulative loading and ligament health (Fig. 1). The general principle that both insufficient and excessive loading may be detrimental is well established across prior frameworks, including hormesis, fitness-fatigue concepts, and Dye’s tissue homeostasis and envelope-of-function models [152–154]. Rather than introducing a new generic dose–response theory, the purpose of this framework is to apply and extend these ideas to the ACL by integrating ligament mechanobiology, movement-specific loading, and recovery-dependent repair processes.

In this ACL-specific interpretation, the key issue is not simply how much load is applied, but whether the tissue is exposed to loading that is sufficient to stimulate adaptation without exceeding its capacity for matrix maintenance and repair. This distinction is especially relevant for the ACL because ligament loading is highly task-dependent, multiplanar, and strongly modified by joint kinematics, neuromuscular control, biological maturation, and recovery status. Emerging mechanobiology evidence further suggests that equivalent fatigue loading may not produce equivalent biological consequences across sexes. In an adolescent mouse model, female ACLs accumulated more collagen denaturation and exhibited a slower, more regulated reparative response than males after equivalent submaximal fatigue loading, whereas males demonstrated a more proliferative and migratory response profile [50]. These findings support the possibility that the transition from adaptive loading to overload may depend not only on external load magnitude or frequency, but also on the tempo of tissue recovery and the timing of reloading.

*Low-load zone:* At the underloading end of the continuum, ligament tissue is exposed to insufficient strain to stimulate meaningful collagen turnover, matrix remodelling, or structural adaptation. This state may arise in sedentary youth, during immobilisation, or during rehabilitation phases in which loading remains overly conservative for prolonged periods. Within this zone, the ACL may remain underprepared for the tensile, shear, and rotational forces encountered during sport, with insufficient stimulus for favourable changes in cross-sectional area, matrix organisation, or mechanical competence. In this sense, the low-load zone reflects a loading environment below the minimum threshold required to support tissue homeostasis and adaptation [152, 154].

*Adaptive zone:* Further along this continuum lies an adaptive range in which appropriately dosed loading, combined with sufficient recovery, may promote favourable ligament adaptation. Within this zone, controlled force transmission is proposed to activate mechanosensitive pathways associated with matrix regulation and tissue maintenance, including factors such as scleraxis, mohawk homeobox, and early growth response 1. From an applied perspective, this zone is characterised not only by progressive overload, but also by loading conditions that limit excessive anterior shear, valgus collapse, and poorly controlled rotational stress during weight-bearing tasks. Thus, for the ACL, adaptive loading should be viewed as a combined function of load magnitude, loading frequency, movement quality, and recovery opportunity, rather than as a simple scalar dose [153, 154].

*Overload zone*: At the opposite end of the curve, chronic overuse, inadequate recovery, or poor neuromuscular control may create conditions in which cumulative micro-damage outpaces repair. Repeated exposure to high-frequency or poorly controlled multiplanar loading may contribute to collagen denaturation, disruption of crimp architecture, and progressive weakening of tissue integrity. Within this framework, overload is conceptualised not only as excessive cumulative loading per se, but also as reloading before adequate matrix recovery has occurred. This distinction is important because it links the framework more directly to emerging ACL mechanobiology: recent experimental findings suggest that female ACL tissue may sustain greater matrix damage and recover more slowly than male tissue under equivalent fatigue loading, raising the possibility that overload thresholds may vary according to sex, maturation, and recovery kinetics [50].

Within this framework, muscle function operates both as an adaptive stimulus and as a protective mechanism. Strain transmitted through the ligament may drive adaptation and contribute to mechanobiological signalling, whereas coordinated muscular action redistributes joint loads and may reduce ACL strain by buffering anterior tibial translation and limiting excessive frontal- and transverse-plane loading. The hamstrings, plantar flexors, and hip abductors appear especially relevant in this protective role. In contrast, quadriceps strength remains essential for performance, but its development must be considered alongside knee angle, landing strategy, posterior-chain contribution, and overall movement organisation so that performance gains are not accompanied by an increase in ACL loading exposure. Accordingly, this framework should be interpreted as an ACL-specific synthesis of established dose–response principles rather than a wholly novel theoretical model. Its potential value lies in linking prior concepts from hormesis, tissue homeostasis, and training theory to the distinctive biological and mechanical context of the ACL. Specifically, it proposes that the boundary between underloading, adaptation, and overload is shaped not only by cumulative mechanical exposure, but also by movement mechanics, biological maturation, sex-specific tissue responses, and the time course of matrix repair. These boundaries remain empirically unresolved, and future work should aim to define load thresholds, recovery windows, and biomechanical conditions that can operationalise this framework for injury prevention, rehabilitation, and return-to-sport decision-making.

## Future Directions

Despite advances in imaging, modelling, and experimental research, key gaps remain in understanding how the ACL adapts to mechanical loading. Longitudinal imaging studies that track cohorts from pre-puberty through PHV are needed to clarify the causal relationship between loading history, maturational timing, and ligament growth trajectories. Such work would directly address the developmental mismatch between rising load demands and relatively fixed ligament capacity that emerges during adolescence. Alongside structural monitoring, the incorporation of in-vivo strain estimation through wearable inertial and electromyography sensors, calibrated against musculoskeletal models, could provide cycle-by-cycle insight into ligament strain during training and competition. This may help refine load prescription and ensure athletes remain within adaptive rather than maladaptive zones of the load-response curve. Future work should also aim to establish quantitative strain thresholds and recovery windows to better distinguish adaptive from maladaptive ligament responses. While these approaches are resource-intensive and may present logistical challenges, particularly in youth populations, they are increasingly being explored and may provide the individualised data currently lacking in ACL prehabilitation frameworks. In parallel, the application of machine learning techniques may allow for down-selection of key predictors into field-friendly, measurable variables, improving translation into applied settings.

Future work must also account for sex-specific biological influences on adaptation. Integrating endocrine profiles with mechanobiological responses may help identify optimal dosing windows for training, such as tailoring strength sessions to hormonal fluctuations in female athletes. Similarly, combined tissue models that couple ligament, tendon, muscle, and cartilage mechanics could predict how altering one structure, for example, hypertrophying or strengthening the hamstrings, redistributes joint loads across the system. Together, these avenues represent the next step in bridging mechanobiological insights with clinical application.

## Conclusion

This review underscores that the ACL is not a passive cord but rather a mechanoresponsive structure capable of hypertrophy, matrix remodelling, and altered tensile behaviour when exposed to judicious mechanical stimuli. Evidence from animal models, imaging, and observational cohorts demonstrates that progressive and well-timed loading, particularly during adolescence, can enlarge ligament CSA and improve material quality, provided muscular supports and neuromuscular strategies distribute forces appropriately. Conversely, underloading leaves the ACL undersized and ill-prepared for athletic demands, while poorly directed or chaotic overload fosters cumulative micro-damage and fatigue failure.

Baseline morphology assessed through advanced imaging, such as MRI-derived measures of length, width, and CSA, provides an important foundation for individualising training. Establishing these structural profiles allows clinicians to modulate loading strategies relative to ligament capacity, ensuring that exercise prescription enhances adaptation without exceeding mechanical tolerance. Effective prevention and rehabilitation must therefore align exercise prescription with ligament biology. High-intensity resistance training restores tissue capacity, while targeted strengthening of the hamstrings, soleus, and hip abductors reduces ACL loading during dynamic and weight-bearing tasks. Neuromuscular strategies such as high-variability training and external focus cues improve the robustness of movement mechanics under unpredictable sport conditions. Programmes must also be individualised, accounting for pubertal stage, sex-specific influences, and notch geometry, to match the biological context in which loading occurs.

By integrating these principles, clinicians and coaches can shift athletes into the adaptive zone of the load-response continuum, simultaneously enhancing performance and safeguarding long-term knee health. Such an approach reframes prevention and rehabilitation as proactive strategies for ligament development, rather than reactive responses to injury. This perspective outlines the importance of aligning training science with ligament biology to secure both short-term performance gains and long-term joint resilience.

## Declarations

### Data Availability

No datasets were generated or analysed for this narrative review.
